# Accuracy of prediction from multi-environment trials for new locations using pedigree information and environmental covariates: the case of sorghum (*Sorghum bicolor* (*L.*)* Moench*) breeding

**DOI:** 10.1007/s00122-024-04684-z

**Published:** 2024-07-10

**Authors:** Diriba Tadese, Hans‑Peter Piepho, Jens Hartung

**Affiliations:** Biostatistics Unit, Institute of Crop Science, University of Hohenheim, Fruwirthstraße 23, 70599 Stuttgart, Germany

## Abstract

**Key messages:**

We investigate a method of extracting and fitting synthetic environmental covariates and pedigree information in multilocation trial data analysis to predict genotype performances in untested locations.

**Abstract:**

Plant breeding trials are usually conducted across multiple testing locations to predict genotype performances in the targeted population of environments. The predictive accuracy can be increased by the use of adequate statistical models. We compared linear mixed models with and without synthetic covariates (SCs) and pedigree information under the identity, the diagonal and the factor-analytic variance-covariance structures of the genotype-by-location interactions. A comparison was made to evaluate the accuracy of different models in predicting genotype performances in untested locations using the mean squared error of predicted differences (MSEPD) and the Spearman rank correlation between predicted and adjusted means. A multi-environmental trial (MET) dataset evaluated for yield performance in the dry lowland sorghum (*Sorghum bicolor* (*L.*) *Moench*) breeding program of Ethiopia was used. For validating our models, we followed a leave-one-location-out cross-validation strategy. A total of 65 environmental covariates (ECs) obtained from the sorghum test locations were considered. The SCs were extracted from the ECs using multivariate partial least squares analysis and subsequently fitted in the linear mixed model. Then, the model was extended accounting for pedigree information. According to the MSEPD, models accounting for SC improve predictive accuracy of genotype performances in the three of the variance-covariance structures compared to others without SC. The rank correlation was also higher for the model with the SC. When the SC was fitted, the rank correlation was 0.58 for the factor analytic, 0.51 for the diagonal and 0.46 for the identity variance-covariance structures. Our approach indicates improvement in predictive accuracy with SC in the context of genotype-by-location interactions of a sorghum breeding in Ethiopia.

**Supplementary Information:**

The online version contains supplementary material available at 10.1007/s00122-024-04684-z.

## Introduction

In plant breeding, genotypic selection for a given target population of environments (TPEs) involves testing of several genotypes across multiple environments and therefore across locations and/or years (Piepho 1998a, b). Different statistical approaches for multi-environment trial (MET) data analysis were proposed over time to increase prediction precision of genotype performances accounting for genotype-by-environment interaction (GEI) effects (Gilmour et al. 1997; Piepho 1998a, b; Smith and Cullis 2018). The common statistical methods used for MET analysis rely on randomization-based models considering different variance–covariance structures for GEI. The simplest variance–covariance structure assumes an identity matrix for the GEI effects, multiplied by a constant variance, implying independence between environments where the genotype main effect is also considered in the model. The identity variance–covariance structure can be replaced by the diagonal and factor-analytic variance–covariance structures for GEI assuming different variances at each environment and/or dependence between environments. The classical approach of modeling MET is based on the fixed effects models (van Eeuwijk 1992; Vargas 1999). Later on, the multiplicative fixed effect model was extended to its random-effects equivalent related to factor-analytic variance–covariance structures in the context of linear mixed model (LMM) (Piepho 1997, 1998a, b; Smith et al. 2001, 2005).

In plant breeding, field trials are usually conducted at a limited number of locations in the TPE, and breeders use statistical methods allowing borrowing information through correlation between genotypes to predict genotypes performances using LMM (Li et al. 2021). In self-pollinated plants, sister lines are correlated through genetic kinship, and the covariance of their breeding values is equal to the additive genetic covariance among the individual lines (Crossa et al. 2006). Therefore, breeders have used methods to incorporate pedigree information and/or marker data in an LMM for predicting breeding values (Buntaran et al. 2022; Crossa et al. 2010; Henderson 1991; Mrode 2005; Pérez-Rodríguez et al. 2015). In the standard LMM, BLUP of breeding values of random genotypes allows borrowing of information among relatives through the coefficient of parentage. Hence, closely related genotypes tend to contribute more to an estimated breeding value than less related lines (Jarquín et al. 2014). Hence, considering the kinship matrix is expected to improve prediction precision of genotypes.

Recent work on MET data analysis has focused on incorporating environmental covariates (ECs) in predicting genotype performances (Li et al. 2021, 2022; Piepho 2022; Piepho and Blancon 2023). In most cases, the main ECs include weather data (for example, rainfall and temperature) and soil information (for example, soil texture, pH, total nitrogen, organic carbon content) are obtained from envirotyping (Xu 2016; Cooper et al. 2014). The ECs can be fitted as regressor variables for the main effects of locations and for genotypes-by-location interactions effects (Li et al. 2021, 2022; Piepho 2022; Resende et al. 2021). Furthermore, breeders may be interested in genotypic prediction for new locations, where the trials were yet not conducted using ECs. Even though the test locations are expected to be representative of the TPE, it can be hard to find a perfect match for new locations among the tested locations. The current advancement in enviromics can improve the selection accuracy across the TPE, including new locations (Resende et al. 2021). This advancement may help to increase prediction accuracy, especially in the case of a limited number of trials.

Often, a large number of ECs is available and regressing the main effect of locations and GEI on several ECs using multiple regression, also known as factorial regression (Denis 1988), may not be practical (Buntaran et al. 2021). One solution for such challenge is to extract a smaller number of synthetic covariates (SCs) that represent the actual ECs (Piepho 2022). In contrast to EC that show only information about a single covariate, SC potentially includes information on all covariates. The extraction of the SCs has been done through a multivariate partial least squares method where the genotype-by-location effects are regressed against the environmental covariates (Piepho and Blancon 2023). Our focus here is to investigate and compare different modeling strategies that provide precise genotypic predictions for untested locations through pedigree information and a large number of ECs. In order to mimic prediction scenarios in new locations, we followed a leave-one-location-out cross-validation (CV) mechanism.

In this study, we propose a modeling strategy for predicting genotype performances in untested locations using MET and evaluate their predictive ability. The general objective was to compare the predictive accuracy of models using MET data analysis (i) without pedigree and SC and (ii) with pedigree or/and SC, under three different variance–covariance structure of the GEI.

## Material and methods

### Data source

We used sorghum (*Sorghum bicolor* (*L.*) *Moench*) data from the Melkasa Agricultural Research Center (MARC), which is located near Adama City, southeast of Addis Ababa. MARC is responsible for the national sorghum breeding program in Ethiopia under the coordination of Ethiopian Institutes of Agricultural Research (EIAR). In this study, we used MET data of the year 2019 which comprises six trials. These trials represent a dry lowland sorghum breeding program of Ethiopia and yield performance of genotypes measured in kilogram per hectare was considered. The field trials were laid out as resolvable row-column designs. In each trial, the plot arrangement was 25 rows by four columns per replicate. One hundred genotypes including two checks (‘Melkam’ and ‘Argiti’) were replicated twice. These genotypes were in fact selected from the previous trials of the ongoing breeding program where 500 genotypes were randomized according to a partially replicated design at three locations. These 100 genotypes were the complete set of genotypes selected from the early breeding stage. Therefore, this dataset is extracted from the ongoing breeding program. All genotypes have pedigree information except for one check (‘Melkam’). Trial’s description and the environmental parameters used in this paper is described in Tables 1 and 2, respectively. The environmental parameters include weather data, and soil information taken at different soil layers. We obtained 65 ECs in total from each trial (Table 2). Most of the soil data were taken from EthioSIS map of 250 m spatial resolution, while some were obtained from ISRIC (FAO 2020). The weather data were obtained from weather stations of sorghum breeding trials in which the average temperature, and rainfall was considered.

**Table 1:** Description of the six dry lowland sorghum breeding trials conducted at six different locations

TrialID	Locations	Longitude	Latitude	Altitude (m.a.s.l)	Minimum T (°C)	Maximum T(°C)	Rainfall (mm)
ER	Erer	42˚15'E	9˚10'N	1323	17	37	778
KB	Kobo	39˚38'E	12˚09'N	1495	14.8	32	678
MH	Mehoni	39˚68'E	12˚51'N	1777	12.81	23.24	539
MS	Mieso	39˚21'E	8˚30'N	1317	16	31	571
SH	Shiraro	39˚90'E	14˚60'N	1034	20.4	34	615
SR	Shewarobit	39˚93'E	10˚35'N	1265	17.7	33	713

*Source* National meteorology, m.a.s.l. = meters above sea level, T = temperature, Rainfall = annual rainfall

**Table 2:** Description of the soil information at different soil layers and weather data taken from each location

Description of the covariates	Layers	Acronyms
Soil organic carbon concentration	6	orc1, orc2, …, orc6
Soil pH	6	ph1, ph2, …, ph6
Coarse fragments volumetric	6	CECrf, Corf, Curf, Ompctrf, CaCO3rf, Mnrt
Soil texture fraction sand	6	sand1, sand2, …, sand6
Soil texture fraction silt	6	silt1, silt2, …, silt6
Soil texture fraction clay	6	clay1, clay2, …, clay6
Cation Exchange Capacity	6	CEC1, CEC2, …, CEC6
Total nitrogen	1	cton
Aluminum concentration	2	MgAIrt, Morf
Exchangeable acidity	3	Prt, pHrt, Npctrt
Exchangeable calcium	2	catomg, Casatrf
Exchangeable magnesium	2	ktomg, Mgsatrf
Exchangeable sodium	2	Srt, Sirt
Sum of exchangeable bases	3	Znrf, ECrf, Krf
Electrical conductivity	6	EC1, EC2, …, EC6
Temperature	–	Temp
Rainfall	–	RF

## Statistical methods

Our modeling strategy follows a stage-wise approach, where information from Stage I is forwarded to Stage II (Piepho et al. 2012). In Stage I, individual locations were subjected to an LMM analysis producing adjusted genotype means and associated variance–covariance matrix of genotype means. Adjusted means and their precision measure from Stage I were then forwarded to the second-stage analysis, where the combined analysis was conducted across locations.

### Stage I analysis

The LMM used for each location in Stage I (Diriba and Piepho 2023) can be expressed as:

1 $$y_{ijkl} = \mu + a_{i} + h_{j} + r_{jk} + c_{jl} + e_{ijkl}$$

where ${y}_{ijkl}$ is the observed yield of the *i*-th genotype in the *k*-th row and *l*-th column within replicate* j*, $\mu$ is the intercept, ${a}_{i}$ is the fixed effect of the *i*-th genotype, ${h}_{j}\sim N(0,{\sigma }_{h}^{2})$ is the random effect of the *j*-th replicate, ${r}_{jk}\sim N(0,{\sigma }_{r}^{2})$ is the random effect of the *k*-th row nested in the *j*-th replicate, ${c}_{jl}\sim N(0,{\sigma }_{c}^{2})$ is the random effect of the *l*-th column nested in the *j*-th replicate and ${e}_{ijkl}\sim N(0,{\sigma }_{e}^{2})$ is the error associated with ${y}_{ijkl}$. Using Eq. (1), we estimated genotype means (${\overline{y} }_{im})$ for the *i*-th (*i* = 1, 2, …, *I* + 1) genotype at each of *M* locations (*m* = 1, 2, …, *M*). To forward information of Stage I analysis, genotype means of the *m*-th location sorted by genotype were put into a vector ${{\varvec{y}}}_{m}=({\overline{y} }_{1m}, {\overline{y} }_{2m}, \dots , {\overline{y} }_{Im})$. Note that only *I* genotype means per location were forwarded to Stage II as standard check ‘Melkam’ was dropped after estimating means in Stage I due to missing pedigree information. Furthermore, we defined the vector ${\varvec{y}}=({{\varvec{y}}}_{1}, {{\varvec{y}}}_{2}, \dots , {{\varvec{y}}}_{M})$ as a vector of genotype means across locations. To forward precision of genotype means, weights were calculated from the Stage I estimated error variance–covariance structure of genotype means ${{\varvec{y}}}_{m}$ denoted as ${{\varvec{\Omega}}}_{m}$. The inverse ${{\varvec{\Omega}}}_{m}^{-1}$ was approximated by a diagonal matrix formed by the diagonal elements of ${{\varvec{\Omega}}}_{m}^{-1}$ (Damesa et al. 2017; Smith et al. 2001). Hence, these diagonal elements were used as weights in the second stage. The matrix with the inverses of the weights down the diagonal approximates ${{\varvec{\Omega}}}_{m}$ and will be denoted as ${{\varvec{\Omega}}}_{m}^{(d)}$.

### Stage II analysis

In Stage II, we considered genotype as the random factor. This allows to include pedigree information. In addition, location was considered as a random factor, too, so that prediction for new locations is possible. Therefore, with vector ${\varvec{a}} = {({a}_{1}, {a}_{2},\dots ,{a}_{I})}^{T}$ of genotype effects, vector ${\varvec{l}}={({l}_{1}, {l}_{2}, \dots , {l}_{M})}^{T}$ of location effects and vector ${\varvec{s}}={({s}_{11}, {s}_{12}, \dots ,{s}_{1M},{s}_{21},{s}_{22}\dots , {s}_{2M},\dots , {s}_{IM})}^{T}$ of genotype-by-location interaction effects, the basic model considered in Stage II analysis can be written as

2 $${\varvec{y}} = {\mathbf{1}}_{IM} \mu + {\varvec{Z}}_{1} a + {\varvec{Z}}_{2} {\varvec{l}} + {\varvec{Z}}_{3} {\varvec{s}} + \user2{f }$$

where ${\varvec{y}}$ is a vector of estimated genotype-by-location means from Stage I, ${1}_{IM}$ is a vectors of ones, $\mu$ is the intercept, ${\varvec{a}}\sim N(0,{\text{I}}_{I}{\sigma }_{a}^{2}), {\varvec{l}}\sim N(0,{\text{I}}_{M}{\sigma }_{l}^{2})$ and ${\varvec{s}}\sim N(0,{\text{I}}_{IM}{\sigma }_{s}^{2})$ are vectors of genotype, location and genotype-by-location interaction parameters where **I** is identity matrix with the subscript represent the dimension, ${{\varvec{Z}}}_{1},\boldsymbol{ }{{\varvec{Z}}}_{2}$ and ${{\varvec{Z}}}_{3}$ are the corresponding design matrices, respectively and ${\varvec{f}}$ is a vector of error containing sub-vectors ${{\varvec{f}}}_{m}$, with var(${{\varvec{f}}}_{m})$**=**${{\varvec{\Omega}}}_{m}^{(d)}$. The total variance–covariance matrix of ${\varvec{f}}$ is given by

$$var\left( {\varvec{f}} \right) = \left( {\begin{array}{*{20}c} {{{\varvec{\Omega}}}_{1}^{\left( d \right)} } & {0 \cdots } & 0 \\ {\begin{array}{*{20}c} 0 \\ \vdots \\ \end{array} } & {\begin{array}{*{20}c} {{{\varvec{\Omega}}}_{2}^{\left( d \right)} } & {} \\ {} & \ddots \\ \end{array} } & \vdots \\ 0 & \cdots & {{{\varvec{\Omega}}}_{M}^{\left( d \right)} } \\ \end{array} } \right) = \oplus_{m = 1}^{M} {{\varvec{\Omega}}}_{m}^{\left( d \right)} = {{\varvec{\Omega}}}^{\left( d \right)}$$

### Modeling covariance structures and pedigree information

The basic model assumes homogeneous variances and independence between genotype, location and genotype-by-location interactions effects, fitting identity matrices with constant variance in the corresponding variance–covariance structures. The baseline model was modified in two ways. First, the identity matrix for the genotype-by-location interactions was modified to allow for diagonal or FA variance–covariance structures. Second, the independence between genotypes was modified to allow for a kinship matrix. The kinship matrix, multiplied by the genetic variance, contains the genetic variances on the diagonal while the off-diagonal elements are the genetic covariance between pairs of genotypes. Therefore, with $\mathbf{\rm A}$ matrix representing the $I\times I$ numerator relationship for $I$ genotypes, the variance–covariance structures in Eq. (2) can be redefined as ${\varvec{a}}\sim N(0,{{\varvec{\Gamma}}\sigma }_{a}^{2})$, ${\varvec{l}}\sim N(0,{\mathbf{I}}_{M}{\sigma }_{l}^{2})$ and ${\varvec{s}}\sim N(0,{\varvec{\Gamma}}\otimes{\varvec{\Pi}})$, where ${\varvec{\Gamma}}=\mathbf{I}$ or $\mathbf{A}$**,** ${\varvec{\Pi}}=\mathbf{I}{\sigma }_{s}^{2}$, ${\varvec{\Pi}}={\varvec{\Phi}}$ (diagonal) or ${\varvec{\Pi}}={\varvec{\Sigma}}$ (FA) with

3 $${{\varvec{\Phi}}} = \left( {\begin{array}{*{20}c} {\sigma_{s1}^{2} } & {0 \cdots } & 0 \\ {\begin{array}{*{20}c} 0 \\ \vdots \\ \end{array} } & {\begin{array}{*{20}c} {\sigma_{s2}^{2} } & {} \\ {} & \ddots \\ \end{array} } & \vdots \\ 0 & \cdots & {\sigma_{sM}^{2} } \\ \end{array} } \right)$$

and $\otimes$ denotes Kronecker product (Burgueño et al 2012).

For the FA structure, multiplicative terms for approximating the variance–covariance matrix of the genotypes-by-location interaction effects (Piepho 1997, 1998a, b; Smith et al. 2001; Crossa et al. 2004, 2006; Burgueño et al. 2011, 2012) were used. In this case, the variance–covariance structure for the genotype-by-location interactions can be expressed as $\text{FA}\left(K\right)={\varvec{\Sigma}}=\left(\wedge {\wedge }^{\boldsymbol{^{\prime}}}+{\varvec{\Phi}}\right)$, where *K* is the number of latent factors, $\wedge$ is the $M\times K$ matrix in which the *k*-th column contains location loadings for *k*-th latent factor and ${\varvec{\Phi}}$ is a $M\times M$ diagonal matrix (Burgueño et al. 2012). When modeling the variance–covariance using FA structures, it is possible to consider more than one component; however, as the number of components gets larger, there can be numerical problems of fitting the model (Studnicki et al. 2017). In order to decide on the number of components, one can use information criteria like the Akaike Information Criterion (AIC) (Wolfinger 1993). In our case, we tried FA(1), FA(2) and FA(3) orders and selected the FA(2) structure based on AIC.

### Modeling environmental covariates

For modeling environmental covariates, we extracted SCs from ECs for each location that represents the actual ECs following a method proposed by Piepho and Blancon (2023). This method extracts a set of SCs from a set of independent variables where the extraction is achieved through a set of orthogonal factors named as latent variables (Krishnan 2010). Before extracting SCs, the ECs were standardized to mean zero and unit variance. Then, the standardized covariates were fitted against genotype-environment means using a multivariate partial least squares (PLS) approach regarding each genotype’s response as a different variates to get the SC. We used the ‘*mvr’* function in R package for extracting SCs.

Then the extension of the model of Eq. (2) when considering the first SC can be written as follows

4 $${\varvec{y}} = 1_{IM} \mu + {\varvec{t}}\beta + {\text{Z}}_{1} {\varvec{a}} + {\text{Z}}_{2} {\varvec{l}} + {\text{Z}}_{3} {\varvec{s}} + {\text{Z}}_{4} {\varvec{b}} + \user2{f }$$

where $\beta$ is the slope of ${\varvec{t}}$, where ${\varvec{t}}$ is a vector of SC of different environments, ${\varvec{a}}$ is a vector of random intercepts and ${\varvec{b}}=({b}_{1}, {b}_{2},\dots , {b}_{I})$ is a vector of random slope effects with ${\text{Z}}_{4}$ is the corresponding design matrix. When allowing for an unstructured variance–covariance matrix for random coefficients ${\varvec{a}}$ for the intercept and ${\varvec{b}}$ for the slope of each genotype, $$\left[\begin{array}{c}{\varvec{a}} \\ {\varvec{b}}\end{array}\right]\sim N(0, {\varvec{G}}\otimes{\varvec{\Gamma}})$$, where $${\varvec{G}}=\left[\begin{array}{cc}{\sigma }_{a}^{2}& {\sigma }_{ab}\\ {\sigma }_{ab}& {\sigma }_{b}^{2}\end{array}\right]$$ and ${\varvec{\Gamma}}$ is either ***I*** or $\mathbf{A}$. An extension to consider several SCs is straightforward, using the intercept ${\varvec{a}}$ and slopes ${{\varvec{b}}}_{1},\boldsymbol{ }{{\varvec{b}}}_{2}, \dots$ for the SCs. The basis for deciding on the number of SCs is to check if additional SC can improve prediction accuracy. The first SC is expected to explain the largest amount of variance compared to subsequent ones. We used coefficients of environmental covariates to identify the dominant variates in the extracted SC. In addition, characterization of environment using ECs was done by a PLS biplot.

Following Eq. (2) to Eq. (4), a series of 12 models in stage II analysis were fitted and compared for predictive accuracy of genotype performances in untested locations as summarized in Table 3 considering the first SC.

**Table 3:** Summary of the 12 models used to predict genotypes performance in the new locations using the first SC and pedigree information

Models	Fixed effects	Random effects	Variance–covariance matrix of		
			${\varvec{a}}$	${\varvec{l}}$	${\varvec{s}}$
M1	$\mu$	${\varvec{a}}, {\varvec{l}},{\varvec{s}}$	${{\mathbf{I}}_{I}\sigma }_{a}^{2}$	${\mathbf{I}}_{M}{\sigma }_{l}^{2}$	${{\mathbf{I}}_{I}\otimes {\mathbf{I}}_{M}\sigma }_{s}^{2}$
M2	$\mu$	${\varvec{a}}, {\varvec{l}},{\varvec{s}}$	${\mathbf{A}\sigma }_{a}^{2}$	${{\mathbf{I}}_{M}\sigma }_{l}^{2}$	$\mathbf{A}{\otimes {\mathbf{I}}_{M}\sigma }_{s}^{2}$
M3	$\mu , \beta$	${\varvec{a}},{\varvec{b}},{\varvec{l}},{\varvec{s}}$	$$\left[\begin{array}{c}{\varvec{a}}\\ {\varvec{b}}\end{array}\right]\sim N(0,\boldsymbol{ }{\mathbf{I}}_{I}\otimes {\varvec{G}})\boldsymbol{ }{\varvec{G}}=\left[\begin{array}{cc}{\sigma }_{a}^{2}& {\sigma }_{ab}\\ {\sigma }_{ab}& {\sigma }_{b}^{2}\end{array}\right]$$	${{\mathbf{I}}_{M}\sigma }_{l}^{2}$	${{\mathbf{I}}_{I}\otimes {\mathbf{I}}_{M}\sigma }_{s}^{2}$
M4	$\mu ,$ $\beta$	${\varvec{a}},{\varvec{b}},{\varvec{l}},{\varvec{s}}$	$$\left[\begin{array}{c}{\varvec{a}}\\ {\varvec{b}}\end{array}\right]\sim N(0,\boldsymbol{ }\mathbf{A}\otimes {\varvec{G}})\boldsymbol{ }{\varvec{G}}=\left[\begin{array}{cc}{\sigma }_{a}^{2}& {\sigma }_{ab}\\ {\sigma }_{ab}& {\sigma }_{b}^{2}\end{array}\right]$$	${{\mathbf{I}}_{M}\sigma }_{l}^{2}$	$\mathbf{A}{\otimes {\mathbf{I}}_{M}\sigma }_{s}^{2}$
M5	$\mu$	${\varvec{a}}, {\varvec{l}},{\varvec{s}}$	${{\mathbf{I}}_{I}\sigma }_{a}^{2}$	${\mathbf{I}}_{M}{\sigma }_{l}^{2}$	${\mathbf{I}}_{I}\otimes{\varvec{\Phi}}$
M6	$\mu$	${\varvec{a}}, {\varvec{l}},{\varvec{s}}$	${\mathbf{A}\sigma }_{a}^{2}$	${{\mathbf{I}}_{M}\sigma }_{l}^{2}$	$\mathbf{A}\otimes{\varvec{\Phi}}$
M7	$\mu ,$ $\beta$	${\varvec{a}},{\varvec{b}},{\varvec{l}},{\varvec{s}}$	$$\left[\begin{array}{c}{\varvec{a}}\\ {\varvec{b}}\end{array}\right]\sim N(0,\boldsymbol{ }{\mathbf{I}}_{I}\otimes {\varvec{G}})\boldsymbol{ }{\varvec{G}}=\left[\begin{array}{cc}{\sigma }_{a}^{2}& {\sigma }_{ab}\\ {\sigma }_{ab}& {\sigma }_{b}^{2}\end{array}\right]$$	${{\mathbf{I}}_{M}\sigma }_{l}^{2}$	${\mathbf{I}}_{I}\otimes{\varvec{\Phi}}$
M8	$\mu$, $\beta$	${\varvec{a}},{\varvec{b}},{\varvec{l}},{\varvec{s}}$	$$\left[\begin{array}{c}{\varvec{a}}\\ {\varvec{b}}\end{array}\right]\sim N(0,\boldsymbol{ }\mathbf{A}\otimes {\varvec{G}})\boldsymbol{ }{\varvec{G}}=\left[\begin{array}{cc}{\sigma }_{a}^{2}& {\sigma }_{ab}\\ {\sigma }_{ab}& {\sigma }_{b}^{2}\end{array}\right]$$	${{\mathbf{I}}_{M}\sigma }_{l}^{2}$	$\mathbf{A}\otimes{\varvec{\Phi}}$
M9	$\mu$	${\varvec{a}}, {\varvec{l}},{\varvec{s}}$	${{\mathbf{I}}_{I}\sigma }_{a}^{2}$	${\mathbf{I}}_{M}{\sigma }_{l}^{2}$	${\mathbf{I}}_{I}\otimes{\varvec{\Sigma}}$
M10	$\mu$	${\varvec{a}}, {\varvec{l}},{\varvec{s}}$	$\mathbf{A}{\sigma }_{a}^{2}$	${{\mathbf{I}}_{M}\sigma }_{l}^{2}$	$\mathbf{A}\otimes{\varvec{\Sigma}}$
M11	$\mu$, $\beta$	${\varvec{a}},{\varvec{b}},{\varvec{l}},{\varvec{s}}$	$$\left[\begin{array}{c}{\varvec{a}}\\ {\varvec{b}}\end{array}\right]\sim N(0,\boldsymbol{ }{\mathbf{I}}_{I}\otimes {\varvec{G}})\boldsymbol{ }{\varvec{G}}=\left[\begin{array}{cc}{\sigma }_{a}^{2}& {\sigma }_{ab}\\ {\sigma }_{ab}& {\sigma }_{b}^{2}\end{array}\right]$$	${{\mathbf{I}}_{M}\sigma }_{l}^{2}$	${ \mathbf{I}}_{I}\otimes{\varvec{\Sigma}}$
M12	$\mu$, $\beta$	${\varvec{a}},{\varvec{b}},{\varvec{l}},{\varvec{s}}$	$$\left[\begin{array}{c}{\varvec{a}}\\ {\varvec{b}}\end{array}\right]\sim N(0,\mathbf{A}\otimes {\varvec{G}})\boldsymbol{ }{\varvec{G}}=\left[\begin{array}{cc}{\sigma }_{a}^{2}& {\sigma }_{ab}\\ {\sigma }_{ab}& {\sigma }_{b}^{2}\end{array}\right]$$	${{\mathbf{I}}_{M}\sigma }_{l}^{2}$	$\mathbf{A}\otimes{\varvec{\Sigma}}$

The matrix **I** represent identity matrix, with the subscript denoting the dimension,${\varvec{\Phi}}$ represents diagonal matrix for the genotype-by-location interactions, $\otimes$ represents the Kronecker product, ${\varvec{\Sigma}}$ is the factor-analytic variance–covariance structure, $\mathbf{\rm A}$ is the kinship matrix, ${\varvec{a}}$ and ${\varvec{b}}$ are vectors of random coefficients for genotypes, ${\varvec{l}}$ is a vector for location main effects, ${\varvec{s}}$ is a vector for genotype-by-location interactions,$\upbeta$ is the slope for the regression on ${\varvec{t}}$, where ${\varvec{t}}$ is the vector of the synthetic covariate, *I* is the number of genotypes, *M* is the number of locations

In Table 3, models M1–M4 were fitted considering the identity variance–covariance structure for the genotype-by-location interactions, M5–M8 were the diagonal and M9–M12 were the FA structures. Within each type of variance–covariance structure, the first model was fitted without SC and pedigree information, the second model with pedigree information, the third model with SC, and the fourth model with SC plus pedigree information.

### Model evaluation

To mimic prediction scenarios in untested locations, a leave-one-location-out CV algorism was implemented. According to this CV, we dropped all genotype means of a given location at a time and assigned means from this location as a validation set whereas the genotype means from the remaining locations served as a training set. A certain limitation of this type of CV is that the prediction accuracy can be affected by population structure and family structure during genomic selection (Xavier 2021; Werner et al. 2020). However, despite this limitation, we think it is the best method for our purpose, which is to assess predictive accuracy for unseen environments. Prediction of genotype means for the dropped location was then made using the training data set. In this study, for the models with SC, the SC from the dropped locations was also considered. The model predictions for the genotypes-by-location means was expected to benefit from borrowing information like between lines in the same location, between genotypes across location and through correlated locations (Burgueño et al. 2012; Buntaran et al. 2021). The prediction of genotype means was computed across locations in all models. When modeling the covariances between locations in the genotype-by-location interactions, like that in the FA structure, predicting genotype means across locations based on the genotype main effect alone can be misleading since part of the genotype main effect may be absorbed in the interactions effect (Piepho and Williams 2024). For this reason, we added the best linear unbiased predictions (BLUPs) of the genotype main effect and average of BLUPs of the interaction effects for the genotype. Therefore, for the FA structure we considered both prediction scenarios. The model accuracy in predicting genotype performances for untested locations was then evaluated and compared.

The model comparison was made using Spearman rank correlation and mean squared errors of prediction differences (MSEPD). The correlation between estimated genotype-by-location means from Stage I and predicted values from Stage II analysis ignoring the corresponding location was computed. The correlation was first computed per location and then, averaged across locations to obtain average correlation for each specific model. The correlation between estimated and predicted values assesses the degree of consistency in genotype ranking (Roostaei et al. 2014). In addition, predictive accuracy of the model was also assessed using MSEPD for genotypes (Buntaran et al. 2021; Studnicki et al. 2017). As proposed by Piepho (1998a, b), the MSEPD focuses on comparing the difference between the observed difference between two genotypes in a given location $\left({\overline{y} }_{im}-{\overline{y} }_{i{\prime}m}\right)$ and the corresponding predicted difference say $\left({z}_{im}-{z}_{i{\prime}m}\right)$. The smallest MSEPD for the differences is an indication of the best model. The MSEPD is computed as

5 $$MSEPD = \frac{{\sum_{m = 1}^{M} \sum_{i = 1}^{I} \sum_{{i^{\prime} \ne i}}^{I} \left[ {\left( {\overline{y}_{im} - \overline{y}_{{i^{\prime}m}} } \right) - \left( {z_{im} - z_{{i^{\prime}m}} } \right)} \right]^{2} }}{{MI\left( {I - 1} \right)}}$$

where *M* is the number of locations, and *I* is the number of genotypes as defined before. In our case, ${z}_{im}$ and ${z}_{i{\prime}m}$ are predicted values obtained from Stage II using data from all locations except of location *m*.

**Fig. 1:**
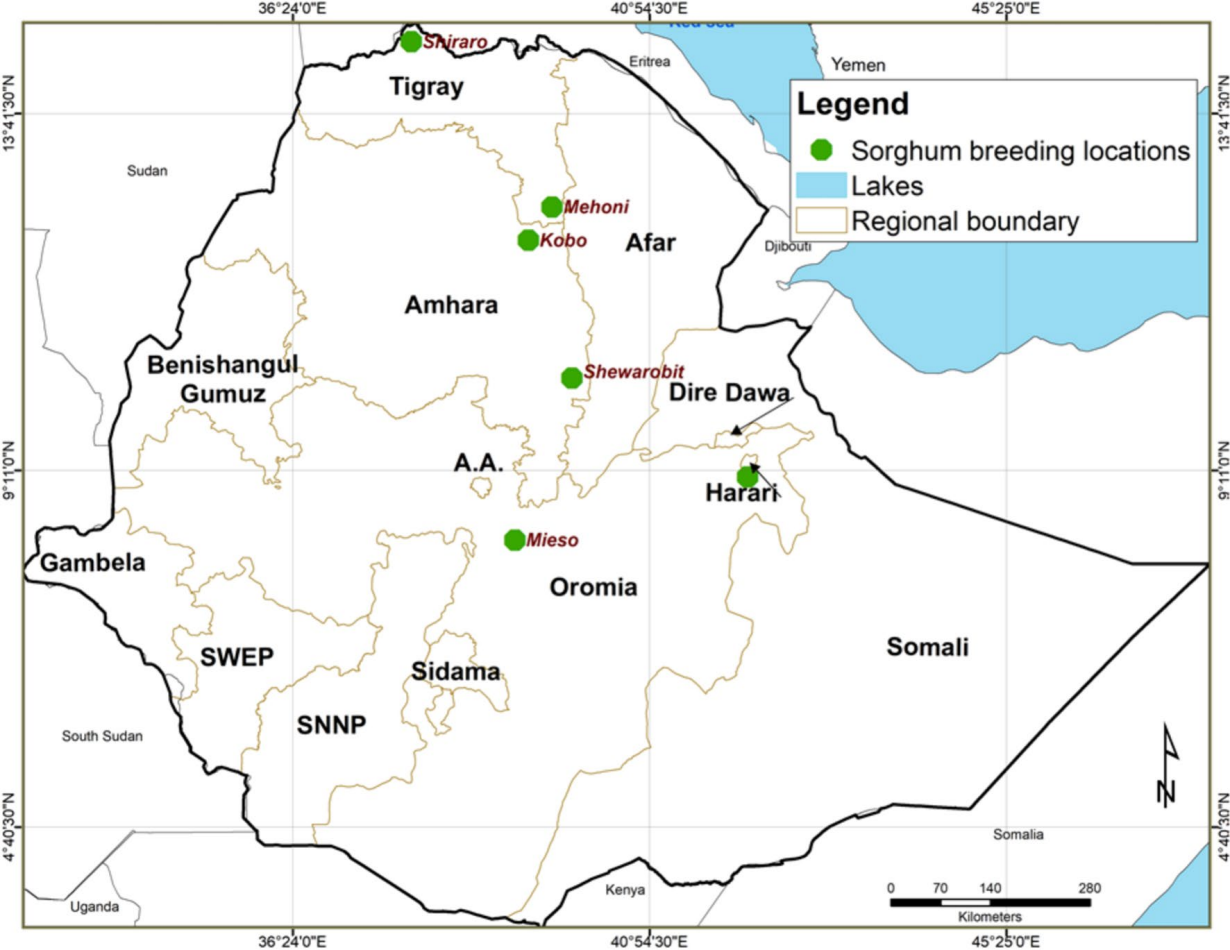
Map of Ethiopia including the six sorghum breeding locations used in this study

We used ASReml-R 4.1.0.130 (Butler et al. 2017) for fitting our models (Fig. 1).

## Results

**Fig. 2:**
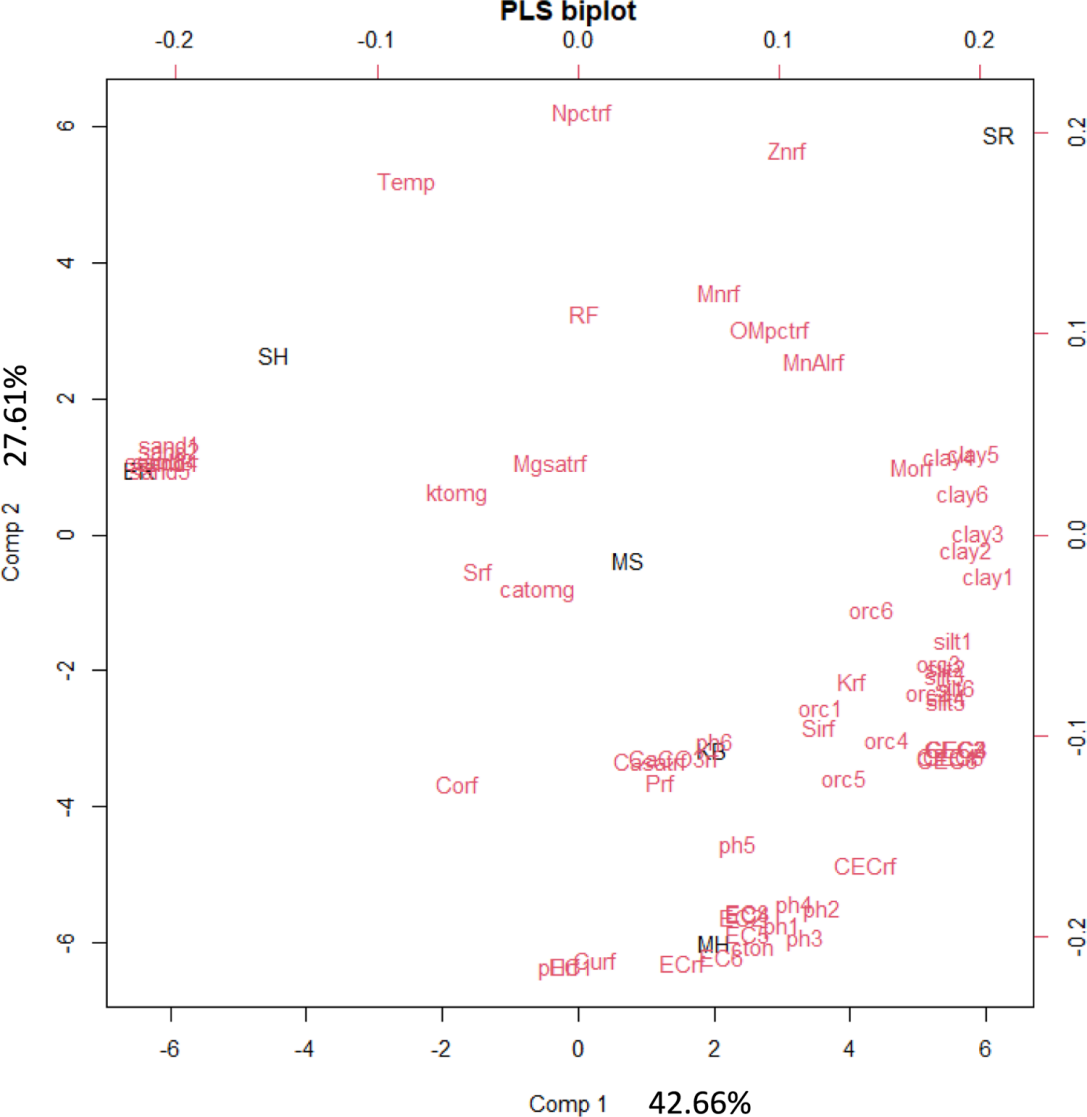
Partial least squares biplot of the environmental variates and environments, where Comp 1 explains 42.66% of the total variance and Comp 2 explains 27.61% of the total variance. For an explanation of the acronyms for locations and environmental covariates, see Tables 1 and 2

Figure 2 shows the PLS-biplot of Comp1 versus Comp2 obtained using the ‘pls’ package in R. In this figure, the first component (Comp1) explains 42.66% of the total variance, while the second component (Comp2) explains 27.61% of the total variance. Both Comp1 and Comp2 jointly explain 70.27% of the total variance.

Table 4 provides rank of the fifteen coefficients of the environmental covariates based on their absolute value. The clay soil property taken at different soil layers is the most dominant in the generated SC. The coefficients of all environmental covariates are provided as a supplementary (S1) file.

**Table 4:** The 15 largest absolute coefficients of environmental variates from multivariate partial least square analysis. Acronyms for the environmental covariates are resolved in Table 2

Rank	Environmental covariates	Coefficients
1	clay5	74.91
2	Znrf	71.65
3	clay4	70.26
4	clay6	68.92
5	clay3	68.23
6	Morf	66.68
7	clay1	66.19
8	sand6	65.52
9	sand5	65.37
10	clay2	64.62
11	sand3	64.08
12	sand4	63.29
13	sand2	61.94
14	sand1	61.33
15	MnAlrf	59.77

Table 5 shows the MSEPD and average rank correlations values of the cross-validation of 12 models. For models without the SC, fitting pedigree data shows some improvement in the diagonal and FA variance–covariance structures of the genotype-by-location interactions when compared to the model without pedigree information. The rank correlation resulted in small difference between models with and without pedigree information in three of the variance–covariance structures. When the first SC was fitted, the diagonal variance–covariance structure resulted in the minimum MSEPD (0.71342 t^2^/ha^2^) followed by the FA (0.90766 t^2^/ha^2^) and the identity (0.95536 t^2^/ha^2^) variance–covariance structures, respectively. Based on the rank correlations, the comparison indicates that the FA variance–covariance structure comes first (0.5837) followed by the diagonal (0.509) and the identity (0.4638) variance–covariance structures. When the first two SCs are considered, there is no gain in the fitted model compared to fitting only one SC for the identity and diagonal variance–covariance structures but a small gain is found with the FA variance–covariance structure according to MSEPD and average rank correlation. With the FA variance–covariance structure, the MSEPD changed from 0.90766 t^2^/ha^2^ when fitting one SC to 0.879093 t^2^/ha^2^ for fitting two SC, whereas the average rank correlation changed from 0.58367 to 0.588384.

**Table 5:** The mean squared error of predicted differences (MSEPD) and average rank correlation between adjusted means and predicted values across locations for each model without SC, with one and two SCs of the cross-validation. The values in brackets in the correlation columns are the standard errors of the correlation for each model across locations

Models	Without SC		With one SC		With two SC	
	MSEPD (t^2^/ha^2^)	Correlation	MSEPD (t^2^/ha^2^)	Correlation	MSEPD (t^2^/ha^2^)	Correlation
M1	1.02248	0.42829 (0.187)	–	–	–	–
M2	1.02311	0.4204 (0.096)	–	–	–	–
M3	–	–	0.95536	0.46375 (0.167)	1.032472	0.425681 (0.098)
M4	–	–	1.02599	0.42105 (0.082)	1.028033	0.420002 (0.083)
M5	1.0464	0.42827 (0.101)	–	–	–	–
M6	1.04629	0.42381 (0.092)	–	–	–	–
M7	–	–	0.71342	0.50913 (0.259)	0.997379	0.47778 (0.168)
M8	–	–	1.0321	0.42548 (0.097)	1.035721	0.423737 (0.089)
M9	1.09375	0.43914 (0.132)	–	–	–	–
M10	1.03831	0.4308 (0.083)	–	–	–	–
M11	–	–	0.90766	0.58367 (0.187)	0.879093	0.588384 (0.196)
M12	–	–	1.00929	0.43857 (0.096)	1.015214	0.437673 (0.097)

M1–M12 are as defined in the Table 3

Table 6 reports the mean squared error of predicted differences (MSEPD) and average rank correlation between adjusted means and predicted values for FA structures with one and without SC obtained by adding BLUP of the genotype main effect and the average of the BLUPs of the interaction effects for genotype. Fitting the SC shows smaller MSEPD and higher rank correlation when compared with others. Table 6 shows higher MSEPD compared with the one in Table 5 for many models and lower rank correlation for the FA structures. The standard errors of the correlations in Table 6 are smaller than those of the ones in Table 5 for all FA structures.

**Table 6:** The mean squared error of predicted differences (MSEPD) and average rank correlation between adjusted means and predicted values for FA model with one and without SC obtained by adding BLUP of the genotype main effect and the average of the BLUPs of the interaction effects for genotype. The values in brackets in the correlation columns are the standard errors of the correlation for each model across locations

Models	MSEPD (t^2^/ha^2^)	Correlation
M9	1.06563	0.42995 (0.087)
M10	1.07402	0.39889 (0.082)
M11	1.02514	0.52711 (0.139)
M12	1.04698	0.41428 (0.088)

M9–M12 are as defined in the Table 3

**Fig. 3:**
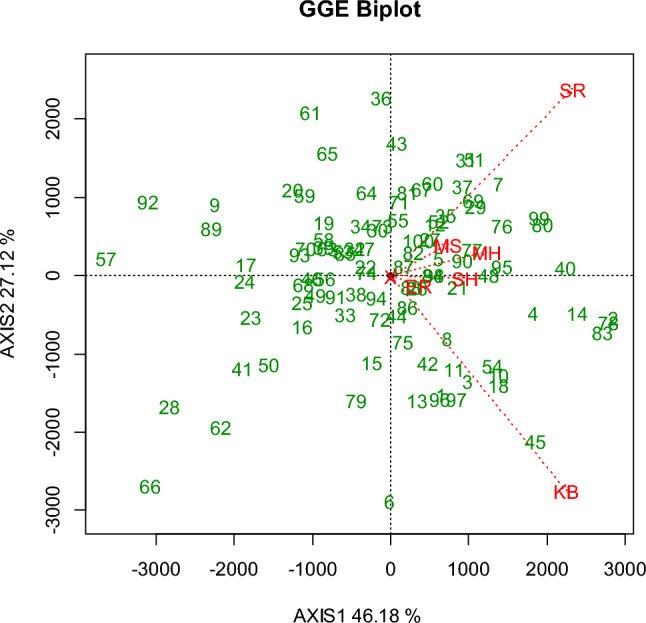
Genotype stability across locations obtained using GGE biplot analysis. The red color represents location names, and the green color represents genotypes. The acronyms for locations are resolved in Table 1

Figure 3 illustrate genotypes stability across locations using GGE biplot analysis. The red lines represent the location names, while the green represent list of genotypes. The plot shows there is a positive correlation between locations. Only ‘KB’ and ‘SR’ seem to be uncorrelated. Genotypes 2, 78, 83 and 14 seem to be good in all locations.

**Fig. 4:**
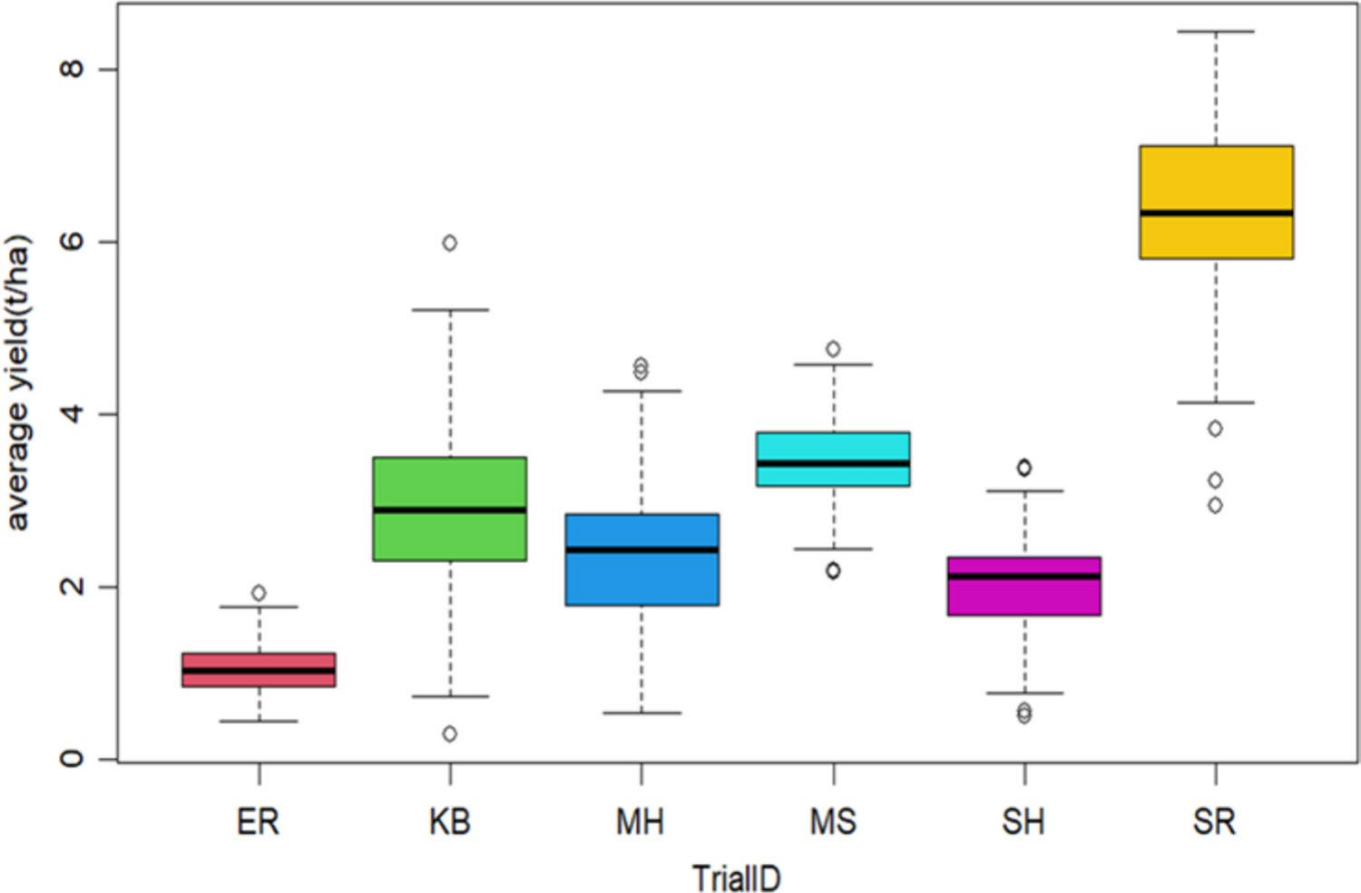
Boxplot plot of mean yield (t/ha) across different locations. The trial IDs are reported in Table 1

Figure 4 shows boxplot of yield response (t/ha) in different locations. On average, the ‘SH’ location shows high yield while ‘ER’ has low yield.

## Discussion

Evaluation of genotype performances in the TPE is a core focus of plant breeders. One advantage of METs is the possibility of allowing borrowing of information among trials during the data analysis (Crossa et al. 2006; Piepho et al. 2008). The MET data analysis can follow either a one-stage or a stage-wise approach for predicting genotype performances in the TPEs. In Stage I of a stage-wise analysis, estimated genotype means and the respective variance–covariance matrix is saved per trial and used during Stage II analysis (Piepho et al. 2012). According to recent studies, a stage-wise analysis has many practical advantages over one-stage analysis (Piepho et al. 2012; Damesa et al. 2017). Three of these advantages are that it is computationally less demanding, that combining trials with different design background is straightforward, and that there are relatively less convergence problems. In our case, the main advantage of the stage-wise analysis was less computational time and fewer convergence problems. During Stage II analysis, different variance–covariance structures of the genotype-by-location interactions can easily be considered with less computational demand to allow for borrowing information through correlated locations. The FA variance–covariance structure is one of the commonly used models in plant breeding (Studnicki et al 2017).

One of the current advancements the plant breeding trials to improve prediction accuracy is incorporating ECs in the data analysis. The main challenge is how to deal with the large number of ECs to fit in the MET. For this purpose, several studies recommended the use of PLS analysis (Vargas et al. 1998, 1999; Crossa et al. 1999; Montesinos-López et al. 2022). It is also possible in principle to consider the actual ECs individually using factorial regression (Denis 1980) with a lower number of ECs; however, with large number of ECs, it is quite difficult to do so (Buntaran et al. 2021; Piepho 2022; Costa-Neto et al. 2023; Piepho and Blancon 2023). In our case, we extracted a smaller number of SCs from the actual ECs using a multivariate PLS technique. This technique considers different genotypes as variates to obtain a single linear combination of covariates to characterize each location (Piepho and Blancon 2023). It also possible to characterize environments through a PLS biplot (Fig. 1).

This study indicated a gain in prediction accuracy from fitting SC in MET compared to the model without SC when predicting genotype means for untested locations, which is also confirmed in other studies (Heslot et al. 2014; Buntaran et al. 2021; Montesinos-López et al. 2022). Jarquín et al. (2014) considered a kinship or kernel regression approach based on ECs to predict genotype performance in an incomplete trial. In our case, we extracted SC from the actual ECs to fit the model using LMM and make predictions. This method is more advantageous compared to fitting the actual ECs since a large number of ECs can be considered through extracting smaller number of SC and the prediction can easily be made for the new locations. Apart from extracting SC through multivariate PLS, different alternative methods of extracting SC were also illustrated in Piepho and Blancon (2023). Here, we favored PLS because it can deal with a larger number of EC. The number and kind of ECs depend on availability and potential of the environmental data. In our case, we consider the soil information more important than weather data since we considered a dry lowland sorghum breeding program, where the temperature and rainfall are relatively similar for all locations. In addition, coefficients of environmental covariates obtained from multivariate PLS analysis help to understand the dominant variates in the extracted SC (Table 4). We would like to stress that the success of GEI modeling depends crucially on the choice of EC and that more is not always better. It is a good strategy to judiciously choose the EC to be included in the analysis based on subject matter knowledge of the crop growth cycle and the key driving factors of growth.

More than one SCs can be fitted in the MET to make prediction. However, the best way is to start with the first SC and check for if there is a gain in fitting more than one SCs. Even if the first SC can capture less than 50%, the first SC capture larger parts of variance compared to all other SCs. Our results showed larger MSEPD and smaller rank correlations with fitting two SCs compared to only fitting the first SC except for FA as indicated in Table 5. We think this is primarily due to the small number of environments. Our results here are based on only six trials and single year data which can be considered as a rather limiting number of environments. However, we believe that a meaningful set of ECs were considered. If available, considering more trials will certainly be advantageous. Fitting two or more SCs in the MET is straightforward using the random coefficients of genotypes for the SCs. When fitting the SC, convergence problems may arise which we approached through setting different initial values of the variance parameters.

The predictive accuracy of pedigree-based models was evaluated, resulting in less gain compared to the one without pedigree and SC. Ideally, pedigree-based modeling allows borrowing strength across genotypes and thereby improving accuracy of genomic prediction. According to recent studies, marker-based models result in more accurate prediction compared to pedigree information (Crossa et al. 2010; Burgueño et al. 2012). Burgueño et al. (2012) compared the prediction accuracy of marker and pedigree-based models. The aim of the comparison was in predicting genotype performances in untested locations using multi-environment mixed models and concluded that marker-based model gives more accurate prediction than pedigree-based model. The basic idea behind fitting either pedigree or marker-based models is that the prediction accuracy is expected to be benefit from correlated information among relatives. In our case, we do not have marker data at hand. The results of fitting pedigree information plus SC are encouraging especially when the diagonal and FA variance–covariance structures for the genotype-by-location interactions were considered.

When different models are used in predicting genotype performances, it is necessary to evaluate the prediction accuracy of the candidate models. When allowing covariances between locations in the genotype-by-location interaction effects, part of the genotype main effect can be absorbed in the interaction effects. In this case, we may need to sum the BLUP of the genotype main effect and average of the BLUPs of the interaction effect for the genotype (Piepho and Williams 2024). The prediction precision of different models can be accessed by using MSEPD as proposed by Piepho (1998a, b) since breeders are more interested in the difference between genotypes means rather than the exact value of specific genotype mean. The model that resulted in the minimum MSEPD is considered as the best model. In our case, the smallest MSEPD was obtained when fitting the SC which highlights the importance of considering ECs for predicting genotype means in the new locations.

In all models, the rank correlation was positive between estimated genotype means and predicted values, with strong correlation obtained for the model with the SC. The strong correlation with the SC is an indication that genotype ranking can be improved through fitting SC. The results for both MSEPD and rank correlation confirm the importance of considering enviromics in genotypic prediction which is also recommended in a recent work (Resende et al. 2024). Particularly, the soil texture (clay and sandy) can be considered as major contributing factor as indicated in Table 4 and needs to be accounted for in the future in the sorghum breeding program.

In this paper, our methods were applied to a single mega-environment; however, the proposed method can also be adopted for more than one mega-environment. With different mega-environments, locations are clustered to form strata or zones where each stratum consisting of several environments (Buntaran et al. 2021). The extension of our proposed methods to several mega-environments is straight-forward, requiring inclusion of a fixed zone effect and its random interactions with genotypes and years in the Stage II analysis. Based on our findings and other recent studies, we conclude that the use of SC increase prediction accuracy in predicting genotype performances for untested locations given that an appropriate statistical model is used (Costa-Neto et al. 2023; Heslot et al. 2014).

## Conclusion

From this study, we conclude that fitting SC can increase prediction accuracy in new locations while the model with both SC plus pedigree information is considered as a promising candidate. The fitted SC performs better in combination with the diagonal and the FA variance–covariance structures of genotype-by-location interactions than when the identity variance–covariance structure was used.

### Supplementary Information

Below is the link to the electronic supplementary material.

Supplementary file1 (ZIP 13 kb)

## Data Availability

The estimated yield data used in Stage II analysis, pedigree and environmetal covariates data are available as electronic Supplementary Materials.

## Code availability

The R code is available as electronic Supplementary Materials.
